# A Role for MAIT Cells in Colorectal Cancer

**DOI:** 10.3389/fimmu.2020.00949

**Published:** 2020-05-20

**Authors:** Stuart P. Berzins, Morgan E. Wallace, George Kannourakis, Jason Kelly

**Affiliations:** ^1^Fiona Elsey Cancer Research Institute, Ballarat, VIC, Australia; ^2^Federation University Australia, Mount Helen, VIC, Australia; ^3^Department of Microbiology and Immunology, Peter Doherty Institute for Infection and Immunity, University of Melbourne, Melbourne, VIC, Australia

**Keywords:** MAIT cells, colorectal cancer, IL-13, tumor immunity, human immunity

## Abstract

MAIT cells are MR1-restricted T cells that are well-known for their anti-microbial properties, but they have recently been associated with different forms of cancer. Several studies have reported activated MAIT cells within the microenvironment of colorectal tumors, but there is conjecture about the nature of their response and whether they are contributing to anti-tumor immunity, or to the progression of the disease. We have reviewed the current state of knowledge about the role of MAIT cells in colorectal cancer, including their likely influence when activated and potential sources of stimulation in the tumor microenvironment. The prospects for MAIT cells being used in clinical settings as biomarkers or as targets of new immunotherapies designed to harness their function are discussed.

## Introduction

Mucosal associated invariant T (MAIT) cells are a unique lineage of unconventional T cells that are important for anti-microbial immunity (1). As their name suggests, MAIT cells are abundant in mucosal tissues, such as gut, lung, and the female genital tract, but they are also well-represented elsewhere, including in the blood, skin, and liver (2–4). Like other unconventional T cells, such as NKT cells and γδ T cells, MAIT cells mount rapid cytokine responses when activated and express cell surface markers that are more commonly associated with NK cells, including CD161 and NKG2D (5, 6). MAIT cells are more abundant in humans than in mice and there is some evidence of a reciprocal relationship between the frequency of MAIT cells and NKT cells, with humans having a relatively high MAIT cell frequency and low NKT cell numbers, whereas the opposite is true for mice (3, 7, 8). This review will focus on human MAIT cells unless otherwise stated.

MAIT cells are restricted by the MHC class 1b molecule MR1 and recognize a narrow array of riboflavin metabolites produced by bacteria and fungi, but not mice or humans (9–11). MAIT cells usually represent 2–5% of human T cells and comprise the most abundant TCR specificity within the antigen-naïve T cell pool (2, 5). This TCR specificity confers the well-documented capacity for MAIT cells to mount potent anti-microbial responses, but they can also become activated in settings that are not directly associated with microbial infections, including respiratory disorders, viral infections, autoimmunity, and cancers (12–19). The nature of MAIT cell involvement in these conditions remains poorly understood, but they can conceivably be activated thorough a bystander response to a proximal microbial infection; through TCR cross-reactivity with structurally similar antigens; or through IL-12 and IL-18 (8, 15, 20). The activation of MAIT cells by innate cytokines is a characteristic they share with NKT cells, which similarly have high expression of IL-12R and IL-18R (20). In this review, we focus on the growing evidence that MAIT cells are influential in anti-tumor immunity against colorectal cancer.

## Colorectal Cancer

MAIT cells have been linked with many different forms of cancer, but the most well-studied association is with colorectal cancer (CRC) (21–24). CRC is one of the most common forms of cancer and continues to present a significant public health burden, so there have been considerable efforts made to develop more effective treatments. Immunotherapies have produced outstanding results in other cancers, but has only been effective against CRC tumors with microsatellite instability caused by mutations of mismatch repair genes. This represents <20% of CRC adenocarcinomas and treatment of the remainder continues to rely heavily on surgical resection of the affected area of the bowel, followed by chemotherapy (25, 26).

CRC usually develops from adenomatous polyps, of which ~5% eventually progress to an invasive carcinoma (27). For the purposes of this review, CRC can be assumed to refer to this group of adenocarcinomas unless otherwise indicated. The formation of polyps and their transformation to malignancy can take many years and there are numerous contributing factors, including the gradual accumulation of genetic and epigenetic mutations, exposure to dietary and environmental carcinogens, and chronic inflammation caused by dysbiosis of the commensal bacterial in the gut, or conditions such as Crohn's disease and ulcerative colitis (27–29). This means that inflammation of this region is associated both with anti-tumor immunity as well as with disease progression, which makes it challenging to dissect the roles of immune cells in the region, including that of MAIT cells (21, 30).

## Barrier Defects in CRC

The epithelial layer of the intestines separates the luminal contents of the gut from the rest of the body (31). The growth of polyps and CRC tumors can cause local failures of epithelial barrier function and other tissue damage which can result in inflammation and predispose to CRC (32, 33). Barrier damage usually induces tissue repair mechanisms such as the IL-18-MYD88 axis, however in CRC, these cannot overcome the ongoing tissue damage caused by neoplastic tumor growth and a setting of chronic barrier impairment and inflammation becomes established (29, 30, 34). This inflammation often coincides with an invasion of commensal bacterial and associated microbial products and these factors collectively promote CRC progression and an immunosuppressive microenvironment (27). For example, crosstalk between commensal bacteria and immune cells can result in the release of IL-6 from myeloid derived suppressor cells (MDSCs) and IL-17 from Th17 cells, which are both potentially tumorigenic (34). Furthermore, many tumor cells release cytokines, chemokines, and growth factors that recruit and induce FOXP3+ Tregs, and induce MDSCs and macrophages to release Th2 cytokines such as Tgf-β, IL-4, IL-10, and IL-13 which suppress anti-tumor immunity (29, 34, 35).

The loss of barrier integrity in CRC is interesting because MAIT cells are abundant in the lamina propria and epithelial layer of the intestine and as such, they are likely to be chronically exposed to bacteria from the gut and to the localized inflammation in this region (4, 22, 36). MAIT cells can therefore potentially become activated through TCR-mediated recognition of metabolites from commensal bacteria that reside in the gut, or independently of antigen recognition through IL-12 and IL-18 (20, 37, 38). It is also possible, but not proven, that MAIT cells could recognize tumor derived antigens (19, 39). As such, CRC and precancerous polyps provide a rich microenvironment for chronic MAIT cell activation.

## T Cells in CRC

Other T cell lineages are also resident in these areas, or will migrate to tumors in response to chronic inflammation. The composition and functioning of T cells in and around tumors are important prognostic indicators for CRC, with infiltration by CD8+ cells and Th1 cytokine responses both regarded as positive prognostic indicators, whereas a high frequency of FOXP3+ Tregs, Th17, and Th2 T cells are often associated with poorer outcomes (35). Th2 cytokines such as IL-4, IL-10, IL-13, and Tgf-β that inhibit anti-tumor responses can also be released by myeloid derived suppressor cells (MDSC) that have been activated by tumor cells (40).

## MAIT Cells in CRC

MAIT cells have been identified in the lamina propria and intra-epithelial regions of the normal gut and colonic polypoid tissue, so it is not surprising that they are found in CRC tumors and neighboring tissues (4, 23, 41). However, it remains unclear what role they are playing and what their importance is in disease progression and in anti-tumor immunity. Analysis of MAIT cells from CRC tumors has provided important information about their activation status and functioning in the tumor microenvironment, but conclusions have varied between studies. It is clear that MAIT cells are present in most CRC tumors, but there is no agreement over whether they are participating in a significant anti-tumor response, or even whether MAIT cell activation is helpful or harmful in that setting (39, 42).

Several studies have found a higher frequency of tumor infiltrating MAIT cells than in surrounding healthy tissue, or in blood of the same donor (21–23). This implies that MAIT cells are proliferating and/or migrating to tumors and that premise was initially supported by evidence of increased CD69 expression among tumor-derived MAIT cells, which suggests these cells were activated, but as the same group later reported, this is also a defining trait of tissue-resident T cells (22, 42). Interestingly, some studies also reported a reduced frequency of MAIT cells in the blood of these patients (21, 24), which the authors suggested may have been due to MAIT cells trafficking from the blood (thus reducing frequency) and localizing in and around tumors (21, 24).

While these findings suggest MAIT cells are responding to tumors, the difference in MAIT cell frequency between tumor sites and that of peripheral blood and surrounding healthy colon tissue was relatively minor in most instances, and some studies reported normal blood frequency (22). In studies where CRC had metastasized to the liver, the frequency of MAIT cells was even lower in the secondary tumors than in the surrounding healthy liver, which suggests the tissue region rather than the tumor itself may be a factor in determining the extent of MAIT cell infiltration (43).

It is also important to note that some reports of increased expression of activation markers by tumor-derived MAIT cells relied on CD69 as measure of activation. CD69 is upregulated following T cell activation, but it can also be constitutively expressed by non-activated tissue resident T cells and it is difficult to be sure whether the CD69+ MAIT cells were long term residents, or cells that had recently migrated from the blood (44).

Groups that have assayed cytokine expression by MAIT cells in CRC tumors have mostly relied on assays of MAIT cells conducted after enzymatic digestion of the tumor fragments. This is necessary to produce the single cell suspensions required for those assays, but it does mean the results are a measure of the potential of the MAIT cells rather than a direct measure of whether the cells were producing cytokines *in situ*. Nevertheless, it is noteworthy that most studies have found that MAIT cells from CRC tumors are capable of cytokine release and cytotoxic functions that could potentially impact on the tumors (22, 42). This seems to exclude the possibility that the cells are defective or anergic and highlights their potential as targets for novel immunotherapies that seek to harness MAIT cell functions. However, there is some disagreement as to whether the magnitude of the MAIT cell cytokine response is muted, particularly in relation to IFNγ, where both normal and reduced responses have been described (21, 22, 42, 43).

Expression of IL-17 has also been detected from blood-derived and tumor infiltrating MAIT cells (21, 22, 24). IL-17 can promote CRC tumor growth (39), but the magnitude of the IL-17 response in tumor derived MAIT cells appears to be relatively weak.

On balance, it is evident that MAIT cells are present in tumors, and that they are capable of cytokine release in response to *in vitro* stimulation. There is evidence of increased frequency and activation compared to MAIT cells in peripheral blood and surrounding healthy colon tissue, but the extent of the increases were sometimes relatively minor. This suggests MAIT cell activation is not extensive in tumors, but it must be acknowledged that the methodologies required to isolate and then assay tumor resident MAIT cells are both time consuming and demanding on cells and may lead to an underestimate of MAIT cell activation and activity.

## Activation and Potential Impact

The semi-invariant TCR of MAIT cells recognizes bacterial metabolites, but as previously mentioned, it is possible for MAIT cells to be stimulated independently of TCR-mediated antigen recognition through IL-12 and IL-18. Both are produced in the colon, although levels are reportedly reduced in CRC tumors (45, 46). Sundstrom et al. and Kelly et al. have proposed that MAIT cells in or near tumors might respond to commensal bacteria that entered the lamina propria of the gut when the epithelial layer was breached by tumor growth (36, 42). Activation could occur through direct recognition of microbial, or potentially tumor, antigens, but IL-12/IL-18 can also mediate TCR-independent activation of MAIT cells.

There is an ongoing debate about whether activated MAIT cells are more likely to promote or inhibit cancer progression (19, 36, 39). In favor of MAIT cells playing a beneficial role is their capacity for potent release of Th1 cytokines such as IFNγ and TNF, which both have well established roles in anti-tumor immunity. MAIT cells can also express perforin and granzyme B and they have a demonstrated capacity to lyse target cells, but it is not known whether they can target tumor cells (22, 42, 46). The counter argument is that chronic exposure to pro-inflammatory cytokines such as TNF and especially IL-17, can promote a tumorigenic microenvironment that is immunosuppressive (29, 32, 46). Supporting the hypothesis that MAIT cells are potentially harmful is the finding that high proportions of MAIT cells in and around tumors was a poor prognostic indicator in CRC (23).

Our group has recently added to this story by demonstrating that MAIT cells can produce high levels of IL-13 and IL-5 when stimulated for more than 3 days, which contrasted with the Th1/Th17 response that dominated the early response (36). This shift could be significant in CRC because while IL-13 is important in tissue repair, it can also promote CRC tumor growth and metastasis, and can inhibit anti-tumor immunity by promoting a Th2-type microenvironment (47, 48).

An intriguing aspect to consider is the emerging evidence that MAIT cells play an important role in tissue repair (49, 50). Tumor related breaches of the epithelial layer of the colon are essentially a wound that won't heal and as such, a self-perpetuating supply of chemical and microbial stimuli could lead to the chronic activation of MAIT cells. As we have shown, this can result in significant IL-13 release, which is a key mediator of tissue repair, but may be detrimental in this setting through IL-13R/STAT6 signaling on tumor cells. Similarly, IL-17 from MAIT cells can promote wound healing but is also tumorigenic.

**Figure 1:**
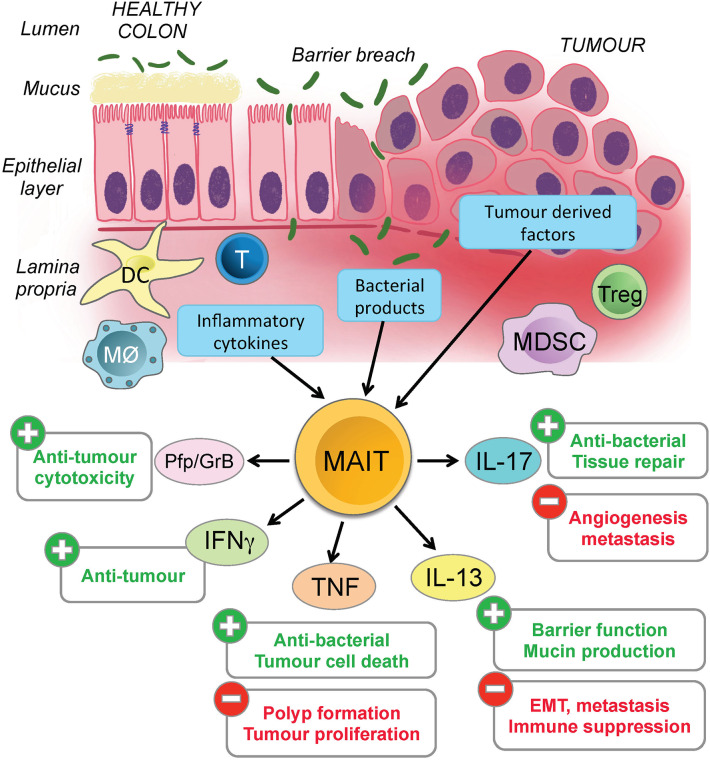
Potential impact of activated MAIT cells in colorectal cancer. Colorectal cancer often results in chronic inflammation and a breach of the epithelial barrier. MAIT cells are found in the lamina propria and could potentially be activated by microbial or tumor antigens, or by inflammatory mediators such cytokines and chemokines released by tumor cells, damaged epithelial cells or various types of immune cells. The figure illustrates the potential ways in which the MAIT cells might become activated (blue boxes) in CRC, the different cytokines they can release and whether these are likely to have a positive or negative effect on the tumor and surrounding microenvironment. Several other important immune cells are represented in the figure, but for clarity, their specific functions and contributions to inflammation in this microenvironment are not detailed.

This raises the possibility that CRC-related breaches of the epithelial barrier directly and indirectly expose MAIT cells to microbial and other inflammatory stimuli from innate immune cells and the inflamed epithelium [(51–53); Figure 1]. Failure to resolve the breach and ongoing inflammation mean that MAIT cells could be chronically stimulated to release Th2 cytokines such as IL-13 that support immune suppression and evasion by CRC cells, thereby explaining the negative association between MAIT cell frequency and therapeutic outcomes. Given this, it is interesting that pre-cancerous polyps and CRC cancer cells both express very high levels of IL-13R and MR1, and our own unpublished studies show they are capable of presenting antigen to MAIT cells (36). The challenge is now to define the nature and consequences of MAIT cells interacting with the CRC tumor microenvironment. This will provide a more complete understanding of CRC progression, and may allow MAIT cells to be exploited for clinical benefit.

## Potential for Clinical Applications

### Prognostic

Several studies have reported that the MAIT cell frequency in blood is reduced in patients with colorectal cancer (21, 24). This observation has also been made in other forms of cancer, perhaps indicating that a MAIT cell deficiency could be considered a risk factor for cancer development (24). If this link is established, screening for MAIT cell frequency could become a useful element of risk assessment during blood screening, however an important caveat is that the reported deficiency is not absolute and is only apparent at a population level when patients are assessed collectively. Indeed, there are studies where patients with cancer have not reported a significant deficiency of MAIT cells in peripheral blood of CRC patients at all (22). When coupled with other confounding factors, such as the fall in MAIT frequency with age, MAIT cell frequency does not appear to be a reliable measure for assessing an individual's risk of CRC (54–56).

### Treatment

A more promising avenue could be the direct targeting of MAIT cells as a treatment for CRC. T cells are often difficult to exploit therapeutically because the diversity of the TCR repertoire prevents effective targeting of specific clones, whereas pan-T cell activation is problematic due to the likelihood of systemic side effects. MAIT cells represent a more attractive target because they express a semi-invariant TCR that is reactive to a well characterized agonist molecule 5-OP-RU. Importantly, a different MR1-restricted molecule, 6-formyl pterin, is a MAIT cell antagonist, so there is potential to activate or block the MAIT cell TCR to promote or prevent cytokine release, depending on which outcome proves to be beneficial (9, 10).

This raises the possibility that MAIT cells could be targeted *in vivo* to selectively activate or inhibit their functions in the tumor microenvironment. This form of treatment could provide a means to boost depleted MAIT cell numbers, or be used to stimulate, modulate or block the cytokine release by activated MAIT cells in the vicinity of the tumor. Clearly, there are many questions to be answered before this is feasible; the most important of which are how MAIT cell activation and cytokine responses are regulated *in vivo*, and whether MAIT cell activation (or suppression) would be clinically beneficial.

The immediate challenge is to develop a fuller understanding of the natural role played by MAIT cells in colorectal cancer, including the profile and consistency of the cytokine response they mount when activated within that microenvironment. While it could previously be assumed that activated MAIT cells would promote Th1 or TH17 cytokine expression, our recent discovery that MAIT cells can produce Th2 cytokines introduces new potential consequences of MAIT cell activation near CRC tumors.

One interesting avenue of recent research has been to investigate whether different forms of stimulation can change the cytokine profile of MAIT cells. This type of functional modulation has been well-described for NKT cells, and there is some evidence of a similar occurrence in MAIT cells (57, 58). It remains uncertain whether this reflects functionally distinct subsets of MAIT cells, or if all MAIT cells can be differentially stimulated to vary their cytokine profile. Our recent study suggests that changing the amount of time MAIT cells are stimulated may also influence the nature of their cytokine response (36). If we can characterize the factors that determine MAIT cell cytokine responses, then it may become possible to exploit the abundance of MAIT cells near colorectal tumors to promote a Th1 response, or to suppress a Th2 response within that microenvironment.

## Unknowns and Future Challenges

It is not yet established whether MAIT cells are producing IL-13 in tumors, but they are present in the tumor microenvironment and accumulate at tumor margins near epithelial breaches, which is consistent with a response to bacterial antigens. It is therefore somewhat surprising that the frequency of MAIT cells and the proportion that are activated is not higher in CRC tumors. Several reports have suggested that a contributing factor to this might be that tumor cells actively inhibit the cytokine response of MAIT cells (59). For example, Sundstrom et al. found that IFNγ production was suppressed when cells were exposed to conditioned media from CRC lines, and Shaler et al. reported metastatic CRC cells in liver suppressed IFNγ production from MAIT cells (22, 43). Our own unpublished studies have also found that some CRC cell lines can inhibit cytokine responses of MAIT cells, so it will be important to determine the specificity and causes of this suppression and whether it occurs *in vivo* in patients.

A critical question is whether antigens are present in the CRC tumor microenvironment that are encountered and recognized by MAIT cells (19). As discussed, it seems likely that resident and infiltrating MAIT cells would be exposed to commensal bacteria when the gut epithelial layer is breached, but it is also important to consider whether tumor antigens might be recognized. One recent study identified an MR1 restricted T cell that could recognize and kill an assortment of autologous and non-autologous cancer cells, while remaining tolerant of healthy cells (60). While this was not a classical MAIT cell, it illustrates the potential significance of related lineages that are restricted by a monomorphic element such as MR1, as these cells are far more likely to have potential pan-cancer activity than HLA-restricted T cells. Given this, and our growing understanding of MAIT cell function, it is imperative to establish whether tumor cells express antigens that are directly recognized by MAIT cells.

Technical issues remain a significant barrier for definitively characterizing the activity of MAIT cells in tumors. MR1 tetramers that enable reliable detection of MAIT cells by flow cytometry are not as effective on tissue sections and *in situ* cytokine profiling remains difficult. Most section staining therefore relies on surrogate MAIT cell markers such as CD161, IL-18R, and CD26, which provide less stringent identification and can introduce unwanted caveats to MAIT cell analysis. The alternative approach of analyzing cell suspensions from digested tumors cannot provide a definitive measure of where individual MAIT cells were localized because ‘tumor samples' typically contain non-tumor tissue types as well. It is likely that the most definitive assessment of MAIT cell location and activity in tumors will rely on analysis of intact tissue, so that MAIT cells from tumor margins, healthy tissue and within tumors can be reliably compared. This will require improved reagents and methodologies to detect and analyse MAIT cells and cytokine profiles.

## Conclusions

It is reasonably certain that colorectal tumors contain MAIT cells that are functionally competent and have the potential to impact CRC through cytokine release and cytotoxicity. Recent studies suggest there may be some pliability in MAIT cell function that provides them with the capacity to be helpful or harmful, depending on the cytokine response they mount and the microenvironment they are in. We have a far better understanding of the potential of MAIT cells to impact CRC, but now face the crucial challenge of determining how distinct MAIT cell responses are triggered and whether the opportunity exists to manipulate them to the advantage of patients with colorectal cancer, and in other diseases where MAIT cells are important.

## Author Contributions

SB and JK planned and researched the article. SB wrote the manuscript. MW prepared the figure. All authors contributed to reviewing and revising the manuscript and approved the final version of the manuscript.

## Conflict of Interest

The authors declare that the research was conducted in the absence of any commercial or financial relationships that could be construed as a potential conflict of interest.
